# Biobanking of human pluripotent stem cells in China

**DOI:** 10.1111/cpr.13180

**Published:** 2022-06-02

**Authors:** Glyn Nigel Stacey, Jie Hao

**Affiliations:** ^1^ National Stem Cell Resource Centre Institute of Zoology Chinese Academy of Sciences Beijing China; ^2^ Innovation Academy for Stem Cell and Regeneration Chinese Academy of Sciences Beijing China; ^3^ International Stem Cell Banking Initiative Barley UK

**Keywords:** cell banking, embryonic stem cells, pluripotent stem cell, regenerative medicine

## Abstract

In recent years, significant progress has been made internationally in the development of human pluripotent stem cell (hPSC)‐derived products for serious and widespread disorders. Biobanking of the cellular starting materials is a crucial component in the delivery of safe and regulatory compliant cell therapies. In China, key players in these developments have been the recently launched National Stem Cell Resource Center (NSCRC) and its partner organizations in Guangzhou and Shanghai who together, have more than 600 hPSC lines formally recorded in the Chinese Ministry of Science and Technology's stem cell registry. In addition, 47 of these hPSCs have also been registered with the hPSCreg project which means they are independently certified for use in European Commission funded research projects. The NSCRC are currently using their own cell lines to manufacture eight different cell types qualified for clinical use, that are being used in nine clinical studies for different indications. The Institute of Zoology at the Chinese Academy of Sciences (IOZ‐CAS) has worked with NSCRC to establish Chinese and international standards in stem cell research. IOZ‐CAS was also a founding partner in the International Stem Cell Banking Initiative which brings together key stem cell banks to agree minimum standards for the provision of pluripotent stem cells for research and clinical use. Here, we describe recent developments in China in the establishment of hPSCs for use in the manufacture of cell therapies and the significant national and international coordination which has now been established to promote the translation of Chinese hPSC‐based products into clinical use according to national and international standards.

## Graphical abstract

The substantial number of pluripotent stem cell lines established in China now have the opportunity to be coordinatedin an initiative involving the Chinese Society for Stem Cell Research, that provides a central resource centre (NSCRC) to facilitate a range of clinical studies all formally recorded in international clinical trials registries.

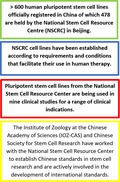

## 1 INTRODUCTION

In recent years, significant progress has been made internationally in the development of human pluripotent stem cell (hPSC)‐derived products for serious and widespread disorders. Biobanking of the cellular starting materials is a crucial component in the delivery of safe and regulatory‐compliant cell therapies. This requires careful documentation of all key stages of the process of procurement of donor cells, cell line generation, biobanking, quality control and release to end users. In recent years, the Chinese regulations related to clinical application of stem cells have been radically revised and provide a new framework for the development of hPSC‐based products.^1^, ^2^, ^3^ The Chinese Society for Stem Cell Biology has also been developing core standards for the use of stem cells, and a key player in the development of these standards has been the Beijing Stem Cell Bank (BSCB). The BSCB was established in 2007 by the Institute of Zoology Chinese Academy of Sciences (IOZ‐CAS). In June 2019, BSCB has redesignated the National Stem Cell Resource Center (NSCRC). NSCRC has worked with partner organizations in Guangzhou and Shanghai to register hPSC lines in the Chinese Ministry of Science and Technology's stem cell registry, and NSCRC has also been developing a collaboration with the European Commission funded database hPSCreg (www.hpscreg.eu).

The NSCRC has now begun to use their human embryonic stem cell (hESC) lines to manufacture eight different cell types qualified for clinical use that are being used in 9 clinical studies for different indications. The IOZ‐CAS was also a founding partner in the International Stem Cell Banking Initiative (www.iscbi.org), which brings together key stem cell banks to agree standards for the provision of pluripotent stem cells for research and clinical use.^4^, ^5^

Here, we describe recent developments in China in the establishment of hPSCs certified for use in cell therapy manufacture to promote the translation of Chinese hPSC‐based products into clinical use according to national and international standards. We also present, for the first time, the recent collaborative work between the Chinese Academy of Sciences NSCRC, the new Institute for Stem Cell and Regeneration (ISCR) and the IOZ‐CAS, that is now driving the establishment of new national and international standards for the use of stem cells. We also describe how these organizations are promoting coordination amongst key national and international stem cell institutions and the approach that the NSCRC is taking to establish the suitability of its cell lines for use in clinical studies.

## 2 HUMAN STEM CELL LINES ESTABLISHED AND VERIFIED IN China

In China, there is a formal process of stem cell line evaluation that is overseen by the Chinese Ministry of Science and Technology which to date has approved over 600 hPSCs. The majority of these hPSCs come from three centres in Beijing (478), Shanghai (119) and Guangzhou (80), each with both hESC and human‐induced pluripotent stem cells (hiPSCs). Furthermore, 47 of these lines have also been registered on the international database hPSCreg (www.hpscreg.eu) which evaluates ethical provenance such as verification of informed consent and scientific data to verify that the cell line is an hPSC. These cell lines, once certified by hPSCreg, are then considered by the European Commission (EC) to be suitable for use in EC‐funded research programmes.

## 3 THE QUALITY AND COMPOSITION OF THE NSCRC CELL STOCKS

Since 2007, NSCRC has established cleanroom facilities and research laboratories which produce hESC lines and approved hESC‐derived product types that are manufactured under a good manufacturing practice (GMP) license from the Chinese Food and Drug Administration (CFDA). NSCRC has operated as a department of the IOZ‐CAS, has produced numerous high‐quality scientific papers on its work in the banking and differentiation of hESC lines for clinical use and plays an important, national and international role in the development of standards in the stem cell area. NSCRC was the first pluripotent stem cell bank in China to be accredited for the supply of cell lines for clinical use under ISO9001:2015 accreditation and has regular GMP inspections by the CFDA. It was also the first biobank in China to successfully pass the Chinese National Accreditation Service (CNAS) inspection against the ISO20387 standard^6^ for general biobanking which was published by the International Standards Organization (ISO) in 2019.^7^

Today, the NSCRC has prepared master cell stocks of more than 350 hESCs, from which those progressing to clinical trial have been independently tested and accredited by the National Institute for Food and Drug Control (NIFDC) for clinical use. The collection as a whole provides cells with diverse human leucocyte antigen (HLA) which potentially could help tissue‐type matching of cell‐based medicines for 70% of HLA‐A, HLA‐B in the Chinese population.

National institute for food and drug control cell lines are checked for documented traceability for 15 different aspects of their preparation covering all stages of cell line development from donor consent, through embryo transfer to NSCRC, to banking and testing prior to release for clinical trials or research work.

All documentation is collated in an individual dossier, known as a ‘Q‐Book’, for each individual cell line to provide a central reference as evidence of their suitability for clinical use. A detailed testing regime for all cell lines intended for clinical use was first published in 2017,^8^ and the testing performed as part of the NSCRC biobanking process is summarized in the bullet point list below.

1. Cell identity (DNA short tandem repeat and isoenzyme analysis).
2. Bacteria, fungi and mycoplasma testing, including endotoxin testing.
3. Testing for specific viruses (human immunodeficiency virus (I and II), human hepatitis virus (B and C), Epstein‐Barr virus, human cytomegalovirus and Treponema Pallidum. (NB additional viral PCR tests have been performed once a cell line is selected for clinical studies such as human T‐lymphotropic virus‐I, human hepatitis A virus, human papillomavirus, human herpes virus (6 and 7), human parvovirus B19 and John Cunningham virus).
4. Testing for general viral contamination was carried out once a cell line was selected for clinical studies, and this has included reverse transcriptase activity, haemagglutination in chick embryo allantoic fluid, survival of inoculated animals (chick embryos, intracerebral and intraperitoneal injection in suckling and adult mice, intraperitoneal injection in Guinea‐pigs, intra‐ and sub‐cutaneous injection in rabbits) and other tests for bovine and porcine viruses.^8^
5. Cell characterization:
  - Typical hESC line morphology in cell culture,
  - Stem cell‐associated markers using immunofluorescence (SSEA‐4, TRA‐1–60, TRA‐1–81, OCT‐4, SOX2 and absence of SSEA‐1) and RT‐PCR (*OCT*‐4, *NANOG*, *SOX*2 and *REX*1),
  - Pluripotency potential by embryoid body differentiation (confirmed by RT‐PCR for production of ectoderm, mesoderm and endoderm specific gene expression and teratoma formation in immunocompromised mice),
  - Karyotype (Geimsa‐banding of metaphase spreads).

All cell characterization reported here was performed by NSCRC staff according to SOPs established under the NSCRC quality management system. The extensive safety testing performed on master stock samples of cell lines selected for clinical studies,^8^ was performed by the NIFDC which is the national centre for independent quality control of stem cell products. The use of NSCRC cell lines in research and clinical applications is reviewed and approved by the IOZ‐CAS Biomedical Ethics Committee and the use of NSCRC cell lines for clinical application is also reviewed and approved by the donor IVF centres. The latter approval procedure is to assure that the intended future use of cells derived from donated embryos is acceptable to all those involved in procurement and development of the cell lines. All cell lines derived at the NSCRC are derived as if they were going to be used to develop cell‐based medicines. As a result, all cell lines, including those latterly allocated for research use, are documented and developed in exactly the same way and in the same facilities, as those taken forward for cell‐based medicine development. Researchers wishing to use NSCRC cell lines in their own research may contact the authors at the NSCRC.

## 4 CLINICAL APPLICATIONS

**FIGURE 1:**
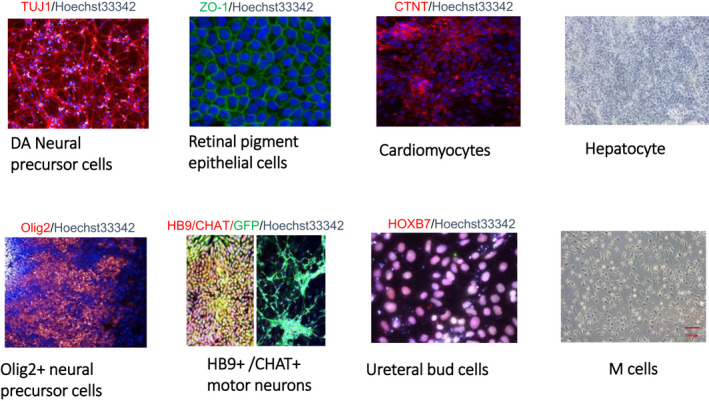
Images of NSCRC hESC‐derived functional cell lines. In the immunostaining panels, the identity of the antibody specificities is marked in the colour (red or green) in which they appear in the respective panel image and co‐staining with nuclear stain Hoechst 33342 was used in all immunostaining. For the motor neuron cell images, GFP staining is shown in the right‐hand panel. The hepatocyte and mesenchymal (M cell) derivatives are shown under phase contrast light microscopy

The NSCRC carries out a range of translational research not discussed here^9^, ^10^, ^11^, ^12^ and has established nine different differentiated hESC product protocols for cell therapy (Figure 1), and each protocol has been independently verified by the NIFDC. In December 2019, there were 69 registered stem cell‐based clinical studies/trials approved by the National Health Commission (NHC) and CFDA. Of these, the majority of hPSC‐based clinical trials (7 of 9) were operated by NSCRC. These included three clinical studies using a mesodermal derivative cell type (‘M Cell’)^13^ for three indications (premature ovarian failure, uterine adhesions and joint meniscus cartilage failure), three clinical studies using a retinal pigmented epithelial cell‐like product^14^, ^15^ for ‘dry’ form of age‐related macula degeneration (AMD) or retinitis pigmentosa (NCT03944239) and one study for Parkinson's disease (NCT03119636).^16^ All of these studies were approved by the CFDA before being registered on clinicaltrials.gov and the WHO clinical trials database. The M Cell products have also been used to treat uterine adhesions^17^ (NCT04232592) and were more recently employed in a number of clinical trials to recover lung function in serious cases of COVID‐19‐induced lung disease (NCT04331613). Translational work is also progressing to develop hESC‐derived hepatocyte‐like cells for clinical treatment of hepatosteatosis.^18^

## 5 STANDARDIZATION ACTIVITY

The NSCRC is playing a very active leading role in the development of biobanking and other bioprocessing standards within the regenerative medicine and biobanking communities. In China, NSCRC actively participated in the Stem Cell Standard Working Group which in the last two years and established two Chinese national standards under the auspices of the Chinese Society for Stem Cell Biology. The first of these standards was for the procurement and general use of stem cells,^19^ and the second was for the technical requirements, test methods, test regulations, instructions for use, labelling, packaging, storage and transportation for hESCs.^20^

The former Director for NSCRC Professor, Qi Zhou, has strongly promoted international standardization in the stem cell field and instigated the development of two ISO standards for pluripotent stem cell biobanking (ISO/WD 24603: 2020 (E)), requirements for the establishment, maintenance, characterization and distribution of mouse and human pluripotent stem cells, and microbiological detection in cell cultures (ISO TC76 WG3 standard in development, general guidance for selection of methods for detection microbiological contamination in mammalian cell cultures). He also was one of the founding members and current chair of the International Stem Cell Forum (ISCF) which formed in 2003 under Professor George Radda at the UK Medical Research Council, to fund collaborations that could only be performed through international coordination. Professor Zhou is also a steering group member of one of the ISCF‐supported programmes called the International Stem Cell Banking Initiative (www.iscbi.org). This activity coordinates consensus amongst experts in pluripotent stem cell banking on key issues for delivery of high‐quality cells for research and clinical application.^4^, ^5^

## 6 SCIENTIFIC COLLABORATIONS AND TRAINING

The NSCRC works with numerous Chinese and international research groups, and NSCRC staff are also engaged in a number of international projects including partnership in the international Parkinson's disease programme G‐Force (http://www.gforce‐pd.com/), advising on the development of the European Biobank for iPSCs (EBiSC) for disease modelling (www.ebisc.org) and as partners in the hPSCreg database of hPSCs (www.hpscreg.eu).

## 7 THE FUTURE

In September 2019, the NSCRC Director Professor, Qi Zhou, organized the inauguration of the National Innovation Alliance of Stem Cell Resource Centers which brings together nine of the most significant cell and stem cell biobanking centres in China to generate a network of scientific excellence sharing standards and establishing a central banking resource for China. A national collaboration on automated stem cell culture is also in development. Furthermore, in addition to its collaboration with hPSCreg (www.hpscreg.eu), NSCRC is also planning to establish a pluripotent stem cell registry for China to coordinate with other registries, such as hPScreg, to promote harmonized procedures and facilitate international communication on stem cell data. China has been highly proactive in its investment in stem cell research and translational research to promote significant benefits for public health, and as part of this plan, NSCRC and IOZ‐CAS look forward to continuing and developing their significant contributions to stem cell research.

## CONFLICT OF INTEREST

The authors have confirmed they have no conflicting interests.

## AUTHOR CONTRIBUTIONS

GNS designed, wrote and revised the manuscript. JH provided data, wrote and revised the manuscript.

## Data Availability

Data sharing not applicable to this article as no datasets were generated or analysed during the current study.
